# Supplementary material for: Content-rich biological network constructed by mining PubMed abstracts
Source: BMC Bioinformatics. 2004 Oct 8;5:147. doi: 10.1186/1471-2105-5-147 (PMC528731; doi:10.1186/1471-2105-5-147)
Supplement: Additional File 2 — The original results of the above study (non-essential files are deleted to keep the file size under the limit set by BMC bioinformatics). [file 1471-2105-5-147-S2.bz2 › chilibotAdditionalFile2/dip05/573ID2164219E10940/html/CD28_CD80.html]

 


 **CD28** and **CD80** 
  
Found 551 abstracts in PubMed, retrieved 05.  
 

 What does Google say? 
 PDF only 
| .edu only 

---

**Interactive relationship** (e.g. stimulation, inhibition, etc)

**Neutral relationship**- The importance of interactions of  **CD80**  CD86 and  **CD28**  in activating CD4 T cells was clear.  Ref: 12794123 J Immunol, 2003
- An effective humoral response requires bidirectional interactions between B cells and CD4 T cells, critical of which are interactions between  **CD80**  CD86 expressed on activated B cells and  **CD28**  expressed on responder CD4 T cells.  Ref: 12794123 J Immunol, 2003

**Non-interactive relationship** (e.g. studied together, co-existance, homology, etc.)

- the ablation of a normal response following the addition of neutralizing anti CD86  **CD80**  Abs mirrored the response of CD4 T cells to B cells of HIV viremic patients, while the addition of exogenous  **CD28**  ligands partially restored the poor CD4 T cell response to the B cells of HIV viremic patients.  Ref: 12794123 J Immunol, 2003
- Melanoma patients with thinner tumors and those surviving revealed an increase of CD19  **CD80**  and CD19  **CD80**  CD86 cells, as well as a higher concentration of CD3 CD4  **CD28**  cells.  Ref: 12878358 Hum ImmunolHum Immunol, 2003
- Changes in the number of  **CD80** , CD86, and  **CD28**  peripheral blood lymphocytes have prognostic value in melanoma patients.  Ref: 12878358 Hum ImmunolHum Immunol, 2003
- We determined the concentration of peripheral blood lymphocytes, which expressed the  **CD80**  CD86 or  **CD28**  CTLA 4 molecules, in 38 patients with cutaneous melanoma and 27 controls.  Ref: 12878358 Hum ImmunolHum Immunol, 2003
